# Effects of Different Drug Therapies and COVID-19 mRNA Vaccination on Semen Quality in a Man with Ankylosing Spondylitis: A Case Report

**DOI:** 10.3390/medicina58020173

**Published:** 2022-01-24

**Authors:** Katerina Chatzimeletiou, Alexandra Fleva, Antonia Sioga, Ioannis Georgiou, Theodoros-Thomas Nikolopoulos, Maria Markopoulou, Nikos Petrogiannis, George Anifandis, Antonios Patrikiou, Efstratios Kolibianakis, Anastasia Giannakou, Grigoris Grimbizis

**Affiliations:** 1 Unit for Human Reproduction, 1st Department of Obstetrics & Gynaecology, ‘Papageorgiou’ General Hospital, Aristotle University Medical School, 56403 Thessaloniki, Greece; thenikdim@auth.gr (T.-T.N.); tonypatr@yahoo.com (A.P.); stratis.kolibianakis@gmail.com (E.K.); grigoris.grimbizis@gmail.com (G.G.); 2 Department of Immunology and Histocompatibility, ‘Papageorgiou’ General Hospital, 56403 Thessaloniki, Greece; alexfleva@gmail.com (A.F.); mariamarkop@hotmail.com (M.M.); giannakouan@gmail.com (A.G.); 3 Laboratory of Histology and Embryology, Aristotle University Medical School, 54124 Thessaloniki, Greece; sioga@auth.gr; 4 Laboratory of Medical Genetics, School of Medicine, University of Ioannina and Medical Genetics and Assisted Reproduction Unit, Department of Obstetrics and Gynecology, University Hospital of Ioannina, 45110 Ioannina, Greece; igeorgio@uoi.gr; 5 IVF Unit, Naval Hospital of Athens, 11521 Athens, Greece; np120@hotmail.com; 6 Department of Obstetrics and Gynecology, School of Health Sciences, Faculty of Medicine, University of Thessaly, 41200 Larisa, Greece; ganif@med.uth.gr

**Keywords:** ankylosing spondylitis, Golimumab, celecoxib, sulphasalazine, mRNA COVID-19 vaccine, sperm, flow cytometry, transmission electron microscopy (TEM), DNA fragmentation

## Abstract

*Background and Objectives:* Ankylosing spondylitis (AS) is a condition that affects 0.1% to 0.5% of the adult population. The aim of this case report was to investigate the possible effects of the drugs taken for treatment of AS as well as mRNA vaccination for COVID-19 on semen quality by performing a highly detailed analysis. *Materials and Methods:* Sperm characteristics were examined by light microscopy, DNA fragmentation (DFI) was analysed by flow cytometry and morphology was evaluated by transmission electron microscopy (TEM). *Results:* Semen analysis under therapy with (1) celecoxib and sulphasalazine showed: concentration 47 million/mL, 53% progressive motility, 7% normal morphology and 9.6% DFI, (2) Golimumab and before mRNA Vaccination showed: concentration 108 million/mL, 82% progressive motility, 1% normal morphology and 7.6% DFI, and (3) Golimumab and after 3 doses of mRNA Vaccination showed: concentration 142 million/mL, 85% progressive motility, 1% normal morphology and 6.8% DFI. TEM revealed head, neck and tail abnormalities, as well as the presence of cells with incomplete spermiogenesis white cells and phagocytes in the sample under therapy with celecoxib and sulphasalazine. Golimumab treatment lead to an increased incidence of elongated heads but in general reduced inflammation as no white cells were evident in TEM. *Conclusion:* The anti-inflamatory drugs celecoxib and sulphasalazine had no adverse effect on sperm quality as all parameters were within normal limits and the patient achieved under that treatment 2 pregnancies following natural conception that lead to the birth of a healthy boy and girl respectively. Anti-TNFa treatment with Golimumab exerted a negative effect on morphology but not on concentration, motility and DFI. After 3 doses of mRNA Vaccination, sperm concentration increased while motility, morphology and DFI remained similar to the values before vaccination suggesting no negative effect of the mRNA vaccine for COVID-19 on sperm quality.

## 1. Introduction

Ankylosing spondylitis (AS) affects 0.1% to 0.5% of the adult population and although it has a genetic predisposition, the development of the disease and severity of symptoms highly depend on environmental factors [1,2]. The HLA-B27 gene, and ERAP1, IL1A, and IL23R have been associated with AS. The pathogenesis of AS lies on the tendency of human leukocytic antigen-B27 heavy chain (HLA-B27 HC) to fold slowly, and in turn, to gradually form a homodimer, with (B27-HC) 2 via a disulfide linkage that activates killer cells, and T-helper 17 cells, inducing endoplasmic reticulum (ER) stress that facilitates triggering of the IL-23/IL-17 axis for pro-inflammatory reactions. [3,4,5,6]

AS patients often have reduced fertility potential, reduced sperm motility, increased frequency of varicocele, increased incidence of sperm aneuploidies, higher plasma LH and FSH, and lower T levels compared to controls [3,5,7,8]. Nukumizu et. al. (2012) [5] reported that AS patients with varicocele have lower sperm quality compared to AS patients without a varicocele. The study notices that a varicocele is more likely to be found in patients with AS and varicocelectomy can improve sperm morphology in these patients. Almeida et al. (2013) [4] reported normal testicular Sertoli cell function in AS patients with mild-to-moderate disease activity, although inflammation in AS appears to be related to impaired testicular function. Various drug therapies have been proposed for the treatment of AS [9,10,11,12,13,14,15,16,17,18,19,20,21,22]. Anti-TNF-α agents seem to be safe on testicular function and fertility and short- and long-term TNF-α blocker therapy does not appear to alter sperm quality [8,9,10,11]. Other drugs commonly used for treatment of AS include celecoxib and sulphasalazine with variable effects on spermatogenesis [13,15,19,21,22,23,24,25].

The effects of the nonsteroidal anti-inflammatory drug celecoxib and the immunosuppressive anti- inflammatory drug sulphasalazine on the patient’s sperm quality has been previously reported in Chatzimeletiou et al. (2018) [23] by analyzing a fresh sample for standard sperm parameters by light microscopy, morphology by transmission electron microscopy (TEM), DNA fragmentation by TUNNEL and chromosomal abnormalities by fluorescence in situ hybridisation (FISH). Here, we extend the previous published data on the patient’s semen quality and present a highly detailed analysis on sperm count, motility, morphology (by both standard light and TEM microscopy) and DNA fragmentation by flow cytometry in a fresh sample collected during the patients current therapy with Golimumab and after full vaccination with three doses of mRNA vaccine and how this compares to his frozen sample cryopreserved during previous therapy with celecoxib and sulphasalazine.

### The Case

A 43-year-old man with AS first came to the Unit for Human Reproduction at Papageorgiou General Hospital in Greece 6 years ago, while under therapy with celecoxib and sulphasalazine, presenting with primary infertility and seeking fertility treatment. The couple had at that time undergone two previous unsuccessful intra-uterine inseminations and one unsuccessful in vitro fertilisation cycle at a different centre. A detailed semen analysis was performed, and sperm cryopreservation followed on two separate occasions as the patient would start in the future a new therapy with Golimumab, a drug that might have had an effect on sperm quality. During detailed investigation, a female factor of infertility was identified and hysteroscopic removal of an endometrial polyp (which was missed during the previous IUI/IVF cycles at a different centre) enabled the subsequent establishment of a pregnancy, following natural conception and the birth of a healthy baby boy. Two years later and while the man still under treatment with celecoxib and sulphasalazine, a second pregnancy following natural conception was achieved that lead to the birth of a healthy baby girl. Now, having completed his family, the man consented his frozen samples to be donated for research and additionally gave a fresh sample before and after mRNA vaccination for COVID-19 (under treatment with Golimumab) to be analysed for all parameters.

## 2. Materials and Methods

A total of 2 cryovials of sperm (cryopreserved during patient therapy with celecoxib and sulphasalazine), which were stored in liquid nitrogen in the Gamete and Embryo Cryopreservation Bank of the Unit for Human Reproduction at Papageorgiou Hospital, Thessaloniki, Greece, were thawed and processed for standard semen analysis. A total of 3.2 mL of fresh semen collected during the patient’s therapy with Golimumab before mRNA vaccination for COVID-19 and 3.5 mL of fresh semen collected during the patient’s current therapy with Golimumab after 3 doses of mRNA vaccination for COVID-19 were processed for standard semen analysis at Fertilia by Genesis, Thessaloniki, Greece. Sperm characteristics were examined by light microscopy, DNA fragmentation was analysed by flow cytometry at the Department of Immunology and Histocompatibility at Papageorgiou Hospital, Thessaloniki, Greece, and morphology was evaluated by light and transmission electron microscopy (TEM) at the Laboratory of Histology and Embryology, Aristotle University Medical School, Thessaloniki Greece. This study was approved by the Bioethics Committee of the Aristotle University Medical School (1.30/21.11.2018) and Genesis (01/7-2/3056). All analysis was performed following the patient’s informed consent.

### 2.1. Standard Semen Analysis

Semen analysis was carried out according to World Health Organization (WHO) criteria: lower reference limits for volume: 1.5 mL; for concentration: 15 millions/mL; for progressive motility A + B: 32%; and for normal morphology: 4% [26].

### 2.2. Transmission Electron Microscopy (TEM)

The fresh and cryopreserved/ thawed sperm samples were resuspended in 3% glutaraldehyde in phosphate buffered saline (PBS) (Sigma-Aldrich Taufkirchen, Germany) (pH 7.4) at 4oC and centrifuged at 1200 rpm for 5 min [23,27]. The supernatant was discarded and the pellet was resuspended in PBS and centrifugations at 1200 rpm for 5 min. The supernatant was then discarded and the pellet was fixed in 1% osmium tetroxide (Sigma-Aldrich Taufkirchen, Germany) for 90 min, washed with PBS and distilled water and stained with 1% aqueous uranyl acetate (Sigma-Aldrich Taufkirchen, Germany) for 14–18 h. The pellet was then dehydrated in 30, 50, 70, 95 and 6 × 100% ethanol series and embedded in Epon 812 (Serve, Heidelberg, Germany). Ultra-thin sections cut in a Reichert ultramicrotome EMUC6 (Leica, Vienna, Austria) were stained with lead citrate (Reynold’s, Merck, Darmstadt, Germany) and were examined on a JEOL TEM 2000 FXII microscope (Jeol, Tokyo, Japan) at 80 KV [23,27].

### 2.3. Flow Cytometry

A total of 100 μL of the fresh and cryopreserved/ thawed sperm samples were washed in PBS (Sigma-Aldrich Taufkirchen, Germany) and centrifuged for 5 min at 300 g. After removal of the supernatant, the precipitant was incubated in TNE buffer (NaCl (0.15Μ), Tris HCL (0.01Μ), EDTA (0.0011Μ) pH 7.4) (Bioline Scientific, Athens, Greece) and detergent solution (NaCl 0,15Μ, TRITON X-100) (Bioline Scientific, Athens, Greece), for 5 min, acridine orange (Bioline Scientific Athens Greece) was added and a further 5 min incubation followed in the dark. The samples were finally analysed by flow cytometry (Beckman Coulter, FC 500, South Kraemer Boulevard Brea, CA, USA) separating the intact sperm (green) from the fragmented ones (red) based on the change in color due to acridine orange inserted in the fragmented portion of sperm.

## 3. Results

**Figure 1:**
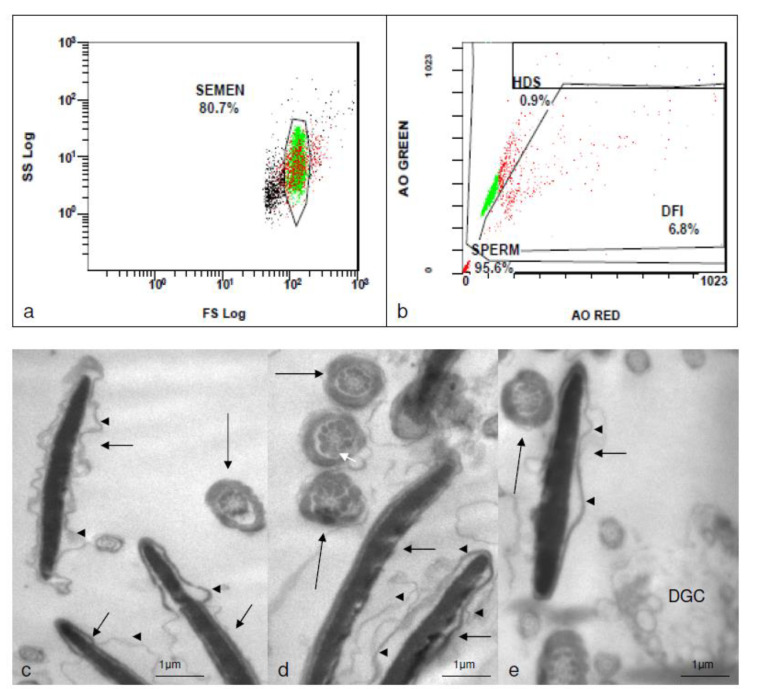
Assessment of DNA fragmentation by flow cytometry (**a**,**b**) and TEM photomicrographs (**c**–**e**) of the fresh sample under therapy with Golimumab and after 3 doses of mRNA vaccine for COVID-19. Note that in (**c**–**e**) the very elongated heads (arrows), the abnormal cytoplasmic membranes (arrow heads), and the normal outer dense fibers and axonemes (long arrows). It is possible that an outer dense fiber is missing in the middle structure (white arrow) in (**d**). Additionally, note the degenerate cell (DGC) in (**e**).

**Figure 2:**
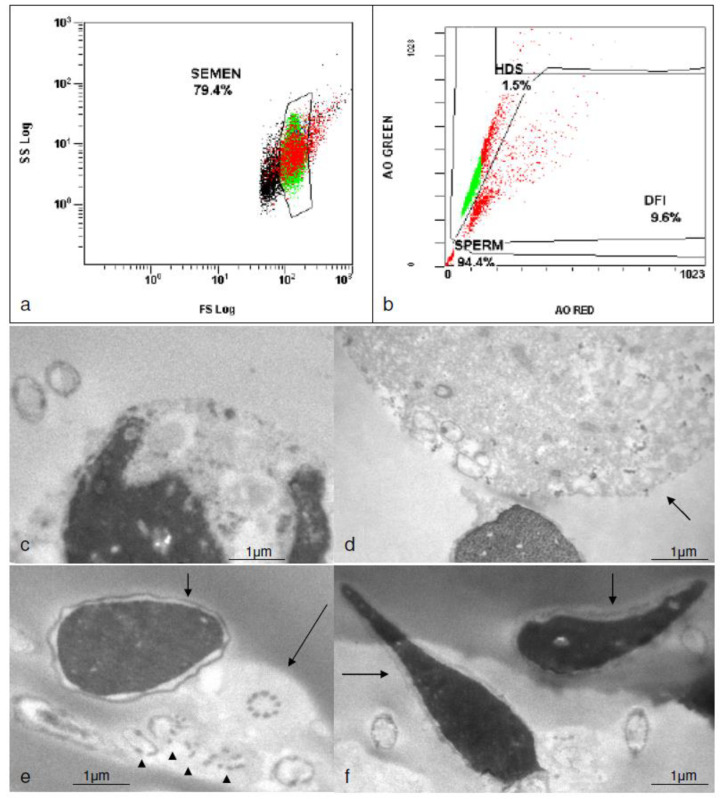
Assessment of DNA fragmentation by flow cytometry (**a**–**b**) and TEM photomicrographs (**c**–**f**) of the frozen sample under therapy with celecoxib and sulphasalazine; note that in (**c**) a neutrophil, in (**d**) a degenerating spermatid (arrow), in (**e**) a longitudinal section of a sperm head with a rounder shape and without a pointed acrosome (small arrow) and cross sections of tails with normal outer dense fibers and axonemal pattern (long arrow) and abnormal patterns (arrow heads), in (**f**) a longitudinal section of a normal sperm head (long arrow) and an abnormally shaped slightly bent sperm head with vacuoles (small arrow).

Standard semen analysis is shown in Table 1. TEM revealed a high incidence of abnormalities on the sperm head (elongated forms) in the fresh samples under current therapy with anti-TNFa (Golimumab) (Figure 1). This was not observed in the frozen sample under therapy with celecoxib and sulphasalazine. Several immature spermatids and several cells with incomplete spermiogenesis as well as phagocytes were however evident in the frozen sample (Figure 2). Sperm DNA fragmentation as assessed by flow cytometry was within the normal limits for all samples tested (9.6% for the frozen sample under therapy with celecoxib and sulphasalazine, 7.6% for the fresh sample under therapy with Golimumab and before mRNA vaccination and 6.8% for the fresh sample under current therapy with Golimumab and after 3 doses of mRNA vaccine (Figure 1 and Figure 2).

## 4. Discussion

The fresh semen sample analysed under current therapy with Golimumab and after mRNA vaccination had all parameters within normal limits according to WHO strict criteria, except morphology, which showed an increased incidence of elongated heads. Golimumab, an anti-TNF monoclonal antibody that minimises immunogenicity, was administered subcutaneously to the patient once a month for the past 4 years. It is unclear why anti-TNFa treatment resulted in a higher incidence of morphologically abnormal spermatozoa, but did not affect concentration, motility and DNA fragmentation, which were all within normal limits. Whether the patient’s genotype for TNF-a or its receptors (TNF-R) is responsible for his semen parameters warrants further investigation. It has previously been shown that TNFR1 36A allele is associated with increased sperm concentration and motility, while TNFR1 36G allele is associated with lower sperm concentration and motility [28,29].

Sperm elongation refers to a sperm head longer than 5 µm and the width <3 µm or a length of <5 µm and a width of <2 µm [30] and is as a stress-induced sperm morphology aberration, prevalent in males with urogenital gland infections or the presence of a varicocele [31]. However, our patient did not have a varicocele and in general followed a healthy lifestyle. Sperm head elongation is associated with severe structural damage as well as severe DNA damage (e.g., chromosomal aneuploidies) [31]. During spermiogenesis, sperm with elongated heads form an abundant complex membranous system between the post-acromial zone and the post-nuclear region [32]. In spermatozoa with elongated heads the acrosome, the post-acrosomal cap and the posterior nuclear space account for 30%, 9% and 6% of the head, respectively, while the posterior part of the head is covered with a voluminous cytoplasmic sheath. The posterior part accounts for 56% of the size of the head [33].

Several mechanisms that lead to elongated sperm heads have been proposed, although only cases with a varicocele and sperm head elongation have been studied. These include a higher scrotal temperature, injured Sertoli cells, wrongly positioned spermatids to Sertoli cells and structurally abnormal Sertoli–germ cell junction complexes. An association between elongated sperm head and an abnormally narrow-shaped spermatid microtubular manchette has been found [33,34]. Previous studies have shown that in mouse models bearing mutations in intra-flagellar transport or microtubule-related proteins (e.g., KIF3A, IFT88, Katanin 80, CEP131, CLIP170 and HOOK1), the manchette is abnormally elongated along with the sperm head [34]. An abnormal manchette exerts excessive pressure on the nucleus before its condensation leading to an elongated sperm head and a tapered nucleus [33]. A significant correlation between abnormal sperm morphology and DNA methyl-transferases (DNMTs) has recently been reported [35] suggesting that morphological abnormalities may influence the distribution of DNMTs leading to changes in DNA methylation, interference with sperm functionality and alterations in cell divisions. Current evidence suggests that patients receiving anti-TNFa therapy may be at an increased risk of developing cancer [36] and, therefore, close monitoring and follow-up testing is recommended.

The cytokine tumor necrosis factor TNFa was initially recognised as one of the products of activated lymphocytes and macrophages secreted in the female pelvic fluids that inhibit and phagocytose spermatozoa [37]. TNFa is also secreted in the male duct in response to chronic inflammation as in spondyloarthritis, supporting the use of anti-TNF medication to prevent sperm abnormalities in such patients [38]. This effect is due to the increase of lipid peroxidation by TNFa and other pro-inflammatory cytokines affecting sperm membranes, during sperm differentiation and sperm tail elongation [39]. Furthermore, human sperm cells have high polyunsaturated fatty acids lipid composition, with high levels of plasmalogenes and sphingomyelins. The specific lipid structure of the sperm membrane underlies the fertilizing properties of sperm, but those lipids are at the same time substrates for peroxidation by ROS [40]. Lipid peroxidation generates lipid aldehydes that directly bind to mitochondrial electron transport chain proteins introducing a vicious cycle with additional ROS imbalance [41]. TNFa also drives the activation of massive signaling kinases surge of tyrosine phosphorylation and sterol oxidation in the sperm tail, and also damages mitochondria in the midpiece, thus affecting tail morphology and function [42]

Vaccination for COVID-19 with 3 doses of mRNA vaccine (Pfizer, BioNTech) also did not exert a negative effect on sperm parameters. In fact, sperm concentration was increased compared to the initial concentration before vaccination and while the man was under treatment with celecoxib and sulphasalazine (Table 1). Our results are in agreement with recent reports confirming no negative alterations in sperm parameters following mRNA vaccination and a significant increase in sperm concentration [43].

The cryopreserved sample under treatment with the anti-inflammatory drugs celecoxib and sulphasalazine had all parameters within normal limits. However, the presence of several immature sperm as revealed by TEM may be in agreement with previous reports suggesting that sulphasalazine may have deleterious effects on spermiogenesis/spermatogenesis [20,21,22,23,24,25]. Sulphasalazine reduces significantly the levels of (B27-HC) on peripheral blood mononuclear cells (PBMCs) including cytokines mRNA levels TNFa, IL-17A, IL-17F IFNγ, and has previously been shown to have a deleterious effect on spermatogenesis and reduce sperm count, motility and morphology. These effects are, however, reversible three months after the removal of the drug [21]. On the other hand, celecoxib, an inhibitor of cyclooxygenase-2 (COX-2; prostaglandin–endoperoxide synthase 2) is constitutively expressed in the testis, where it is responsible for prostaglandin production, so inhibition of this enzyme may have effects on testicular function or ameliorate testicular damage caused by systemic or local inflammation [15,19].

The study’s main limitation is that the patient came to our unit while already under treatment with celecoxib and sulphasalazine and thus it was not possible to have a control sample (under no drug therapy). In addition, there was no gap between the commencement of Golimumab and the discontinuation of celecoxib and sulphasalazine in order to obtain a control sample at that time. Future studies involving a case series of AS patients with available control sperm samples and samples under therapy with different drugs may contribute to a better evaluation of the effects of AS treatment on male fertility.

In the cryopreserved sample under therapy with the anti-inflammatory drugs celecoxib and sulphasalazine, a higher incidence of leukocytes was observed compared to the fresh samples under therapy with Golimumab. This may be attributed to the decrease in inflammation obtained after treatment with the anti-TNFa drug (Golimumab). Leukocytospermia, defined by WHO as >1 × 10^6^ leukocyte /mL, has an incidence of 15% in the general population and although it is especially common in men with infertility, its effects in semen quality is still controversial in the literature [23].

Finally, there is a general concern that SARS-CoV-2 may disturb male fertility, and thus vaccination for COVID-19 to protect from potential infection becomes more and more a necessity [44,45]. Although, the safety of the mRNA vaccines is still under investigation, our results suggest no negative impact on sperm parameters.

## 5. Conclusions

We conclude that for this particular patient the anti-TNFa Golimumab, the anti-inflammatory drugs celecoxib and sulphasalazine and the COVID-19 mRNA vaccine did not exert a negative effect on sperm concentration, motility and DNA fragmentation as they were all within normal limits. Nevertheless, more studies are needed to elucidate the mechanisms leading to alterations in sperm head morphology following anti-TNFa therapy and if this may interfere with sperm’s fertilising capacity or chromosomal constitution. More prospective studies are also needed to evaluate the long-term effects of both the mRNA vaccine and the anti-TNFa and anti-inflammatory drugs on men’s general health and fertility.

## Figures and Tables

**Table 1:** Standard semen analysis.

	Cryopreserved Sperm Sample during Therapy with Celecoxib Sulphasalazine Thawed 6 Years Post-Cryopreservation	Fresh Sperm Sample during Therapy with Golimumab before Vaccination	Fresh Sperm Sample during Current Therapy with Golimumab after Vaccination
Volume	4.3 mL	3.2 mL	3.5 mL
Number/mL	47 × 10^6^/ mL	108 × 10^6^/ mL	142 × 10^6^/ mL
Total Number/ejaculation	202,100,000	345,600,000	497,000,000
**Motility**			
Linear progression	53%	82%	85%
No progression—tail moving	26%	7%	8%
Immotile	21%	11%	7%
**Morphology**			
Normal	7%	1%	1%
Abnormal	93%	99%	99%
Big head	5	1	2
Small head	4	0	0
Long head	4	118	132
Pear shaped head	28	6	5
Round head	1	0	0
Amorphous head	18	11	8
Vacuoles	54	20	15
Small acrosome	2	1	1
Short tail	1	1	1
Double tail	3	1	2
Fourchette	2	1	1
Broken tail	1	1	1
Spiral tail	5	1	1
Asymmetric tail extrusion	1	1	1
Broken neck	14	5	4
Cytoplasmic droplet	32	17	14
Thick mid piece	25	15	12
Round spermatids	7 × 10^6^/ mL	1 × 10^6^/ mL	1 × 10^6^/ mL
White cells	2 × 10^6^/ mL	-	-
DNA fragmentation	9.6%	7.6%	6.8%

## Data Availability

Not acceptable.
